# Effectiveness and safety of Rituximab in multiple sclerosis: an observational study from Southern Switzerland

**DOI:** 10.1371/journal.pone.0197415

**Published:** 2018-05-14

**Authors:** Barbara Scotti, Giulio Disanto, Rosaria Sacco, Marilu’ Guigli, Chiara Zecca, Claudio Gobbi

**Affiliations:** 1 University Hospital Basel, University of Basel, Basel, Switzerland; 2 Neurocentre of Southern Switzerland, Ospedale Civico, Lugano, Switzerland; 3 Università della Svizzera Italiana (USI), Facoltà di scienze biomediche, Lugano, Switzerland; University of Oxford, UNITED KINGDOM

## Abstract

**Background:**

Despite positive results from phase II and observational studies, Rituximab (RTX) is not currently approved for multiple sclerosis (MS) treatment and can only be used off-label.

**Objective:**

To characterize MS patients treated with RTX and investigate its effectiveness and safety in a clinical practice setting.

**Methods:**

Observational analysis of data collected from MS patients at the Neurocenter of Southern Switzerland. Relapses, EDSS worsening, MRI lesion accrual and "evidence of disease activity” (EDA) status were described by Cox regression. RTX and natalizumab treated patients were matched by propensity scores.

**Results:**

Out of 453 MS patients, 82 were treated with RTX, 43 (52.4%) relapsing-remitting (RRMS) and 39 (47.6%) progressive MS (median age = 48 [40–54] years, females n = 60 [73.2%], EDSS = 4.0 [2.5–6.0], median follow-up = 1.5 [1.0–2.5] years). Three relapses occurred and 59 (75.6%) patients had not EDA at follow-up end. Time to EDA was similar in RTX and natalizumab treated RRMS patients (HR = 1.64, 95%CI = 0.46–5.85, p = 0.44). Twenty-four patients presented non infusion related adverse events (infections), requiring RTX discontinuation in 6 individuals.

**Conclusion:**

These results provide further evidence for RTX being effective in MS treatment, to a similar extent to natalizumab in RRMS. Clinicians must be vigilant for the potential occurrence of infections.

## Introduction

Multiple sclerosis (MS) is a chronic immune-mediated inflammatory disease affecting young adults and causing demyelination and neuronal loss in the central nervous system [1]. Several disease-modifying therapies (DMT) are available, including injectable (interferons and glatiramer acetate) [2,3], oral agents, chemotherapeutics (e.g. mitoxantrone) [4] and monoclonal antibodies such as rituximab (RTX), natalizumab (NTZ) and alemtuzumab [5–7].

RTX is a chimeric monoclonal antibody that targets CD20, a glycosylated phosphoprotein expressed on the surface of B cells, leading to their lysis and peripheral depletion. Several studies have shown RTX reduces inflammatory activity, incidence of relapses and new demyelinating lesions on MRI in patients with relapsing-remitting MS (RRMS) [8]. Recent retrospective studies from Sweden have confirmed RTX is highly effective in RRMS, with a discontinuation rate that is lower than that of other DMTs [9]. Similarly to RRMS, there has been suggestive evidence that also patients with progressive MS (PMS) may benefit from RTX treatment, especially those of younger age and with evidence of inflammatory activity on MRI [10]. Available data from RTX studies in MS, but also in other conditions such as rheumatoid arthritis, indicate that RTX is generally well tolerated and safe, even in the long term [11,12].

Despite these encouraging results and its favorable cost-effectiveness profile, RTX is not currently approved for the treatment of MS and can only be administered off-label for this indication. In the absence of a randomized phase III comparative trial, additional observational studies from countries other than Sweden are useful to provide further evidence for RTX use in MS. The aim of this study was to characterize RRMS and PMS patients treated with RTX within a single tertiary care MS center in Southern Switzerland and to investigate its effectiveness and safety, also compared to another highly effective DMT such as NTZ.

## Methods

### Study design and patients

This was a retrospective observational study based on clinical and radiological data that were prospectively and routinely collected within the MS registry of the Neurocenter of Southern Switzerland (Lugano, Switezerland). This represents the only tertiary MS center in the Ticino region, with a population of approximately 400,000 individuals. The registry was started in 2007 and revised in February 2018. Inclusion criteria were: 1) a diagnosis of MS according to McDonald 2010 criteria; 2) having received at least 1 infusion of RTX; 3) available clinical and radiological follow-up data. All patients with neurological conditions other than MS (e.g. neuromyelitis optica, anti-MOG mediated demyelination) were excluded.

### Monitoring of patients and data collection

The time of treatment was defined as the interval between the first RTX infusion (baseline) and the last available neurological examination. Neurological examinations and laboratory tests were routinely performed every 3 months after the first RTX infusion, with neurological disability assessed by the expanded disability status scale (EDSS). All neurologists at the MS center are certified for EDSS assessment (https://www.neurostatus.net/). According to local guidelines, brain MRIs were mandatory at least once a year under RTX treatment, cervical and thoracic spine MRIs were also highly recommended. All brain and spinal (both cervical and thoracic) MRI included in the analysis were performed with a standardized protocol and using 3T scanners (Siemens, Germany) [13].

The following data were collected at baseline: age, gender, disease duration, EDSS, previous MS treatments, reason to switch from other DMT and/or to start RTX, as well as clinical and radiological course in the previous 2 years (i.e. number of relapses, EDSS increase, lesion accrual on brain MRI). The following data were collected at each neurological visit (performed every 3 months): occurrence of relapses, EDSS, occurrence of brain new T2 (NT2) or gadolinium enhancing (GE) lesions, as well as occurrence of infusion related or unrelated adverse events (AE). Laboratory variables were also collected at baseline and every 3 months including total leucocytes, CD19+ B, CD4+ T and CD8+ T cell counts. Similar clinical and radiological information were also collected within the registry on RRMS patients who had received at least one infusion of NTZ between November 2006 and February 2018. All patients who had ever received RTX either before or after NTZ were excluded from this group.

### Statistical analyses

Categorical variables were described by counts and percentages, continuous and ordinal variables by median and interquartile range (IQR). The proportion of patients remaining free of acute relapses, EDSS worsening, NT2 and GE brain lesions during follow-up was estimated for all patients and for RRMS vs PMS using Kaplan-Meier curves. EDSS worsening was defined as an increase in EDSS of ≥1.5 points from an EDSS score of 0.0, ≥1.0 point from an EDSS score of 1.0 to 5.5, or ≥0.5 point from an EDSS score ≥6.0. A similar approach was used to estimate the proportion of patients with “evidence of disease activity” (EDA) status by using relapses, EDSS worsening and MRI lesion accrual (either NT2 or GE lesions) in a single combined outcome. The following variables were tested for association with time EDA by using univariate and multivariate Cox regression models: age, sex, previous treatment with NTZ, baseline EDSS and number of relapses in the 2 years preceding RTX treatment. Patients under treatment with RTX were finally matched in a 1:1 ratio to patients under treatment with NTZ using nearest neighbour matching within a caliper size of 0.1 standard deviations of the logit of the propensity score. This was based on age, sex, EDSS at baseline, relapses in the 2 years before starting treatment and brain T2 lesion load (1–9 vs >9) on baseline MRI scans. All analyses were performed using the statistical software R 3.4.1 GUI 1.70 El Capitan build (7375) and the R packages “*rms*” and “*nonrandom*”.

### Ethical conduct

This is an analysis of data from daily clinical practice. All included patients signed an informed consent for the use of their clinical data for scientific purposes. There is no legal requirement to further register this project in a public registry. In this research work the participant's right to privacy is strictly respected. All data were anonymized before being accessed and analysed.

## Results

### Baseline characteristics

A total of 453 MS patients were included in the MS registry at 31.01.2018. Out of these, 82 (18.1%) had received at least one RTX infusion between September 09.2009 and February 2018. Demographic and baseline characteristics are presented in Table 1. There were 43 (52.4%) RRMS and 39 (47.6%) PMS cases (27 with secondary and 12 with primary progressive disease). The most common DMTs used before RTX were NTZ in RRMS (n = 14, 32.6%) and fingolimod in PMS (n = 9, 23.1%). Median baseline EDSS was 4.0 (2.5–6.0) overall, 2.5 (2.0–3.5) in RRMS and 5.5 (4.0–6.0) in PMS. RRMS patients had more relapses in the two years preceding RTX than PMS (21 vs 5 respectively). Main reasons to switch from other DMTs to RTX in RRMS were disease progression (occurrence of relapses, EDSS worsening or NT2 MRI lesions) (n = 15, 36.6%), anti-JCV antibodies under NTZ treatment (n = 13, 31.7%), and side effects of previous therapies (n = 8, 19.5%). Main reasons to switch to RTX in PMS patients were disease progression (n = 21, 67.7%) and side effects of previous DMTs (n = 5, 16.1%).

**Table 1:** Baseline characteristics at first RTX infusion in all MS, RRMS and PMS patients.

Variables		All patients (n = 82)	RRMS (n = 43)	PMS (n = 39)
**Age**	** **	48 (40–54)	44 (33–51)	51 (45–55)
**Sex**	M	22 (26.8)	11 (25.6)	11 (28.2)
	F	60 (73.2)	32 (74.4)	28 (71.8)
**EDSS**		4 (2.5–6.0)	2.5 (2–3.5.0)	5.5 (4.0–6.0)
**Relapses 2 yrs pre-RTX**	0	56 (68.3)	22 (51.1)	34 (87.2)
	1	21 (25.6)	16 (37.2)	5 (12.8)
	2	3 (3.7)	3 (7.0)	0 (0)
	4	2 (2.4)	2 (4.7)	0 (0)
**Brain T2 lesions**	0–1	1 (1.2)	0 (0)	1 (2.6)
	1–9	22 (26.8)	11 (25.6)	11 (28.2)
	>9	59 (72.0)	32 (74.4)	27 (69.2)
**Brain GE lesions**	No	69 (84.1)	34 (79.1)	35 (89.7)
	Yes	13 (15.9)	9 (20.9)	4 (10.3)
**Spinal T2 lesions**	No	5 (6.5)	5 (12.8)	0 (0)
	Yes	72 (93.5)	34 (87.2)	38 (100.0)
**Spinal GE lesions**	No	74 (96.1)	36 (92.3)	38 (100.0)
	Yes	3 (3.9)	3 (7.7)	0 (0)
**Last DMT**	NTZ	16 (19.5)	14 (32.6)	2 (5.1)
	Fingolimod	18 (22.0)	9 (20.9)	9 (23.1)
	Interferons	11 (13.4)	6 (13.9)	5 (12.8)
	Glatiramer acetate	10 (12.2)	7 (16.3)	3 (7.7)
	Other	17 (20.7)	5 (11.6)	12 (30.8)
	None	10 (12.2)	2 (4.7)	8 (20.5)
**Reason to switch from other DMT**	anti-JCV antibodies	15 (20.8)	13 (31.7)	2 (6.5)
	disease progression	36 (50.0)	15 (36.6)	21(67.7)
	DMTs side effects	13 (18.1)	8 (19.5)	5 (16.1)
	disease progression and side effects	5 (6.9)	2 (4.9)	3 (9.7)
	pregnancy research	3 (4.2)	3 (7.3)	

Continuous and ordinal variables are described by median (IQR), categorical variables by counts and percentage.

### Treatment schedule and follow-up

All patients underwent an initial RTX induction regimen consisting of one infusion on day 1 and a second infusion on day 15 (1,000 mg each in 74 [91.4%] and 500 mg each in 7 [8.6%] patients). A maintenance regimen was then initiated, consisting of a third RTX infusion at 9 months after induction and every 6 months thereafter. The initial maintenance dose after induction was 1,000 mg in 72 (87.8%) and 500 mg in 10 (12.2%) patients, and 27 patients were switched from 1000 mg to 500 mg during follow-up. RTX infusions were performed over a median follow-up of 1.5 (1.0–2.5), with a minimum and maximum follow-up length of 0.5 and 8.3 years, respectively. The first RTX infusion was always preceded by a premedication including iv methylprednisolone 125 mg, iv clemastine 4 mg and oral paracetamol 1 gr. Only clemastine 2 mg and paracetamol 1 gr were administered before subsequent RTX infusions.

### Clinical and radiological effectiveness of RTX in RRMS and PMS patients

**Fig 1:**
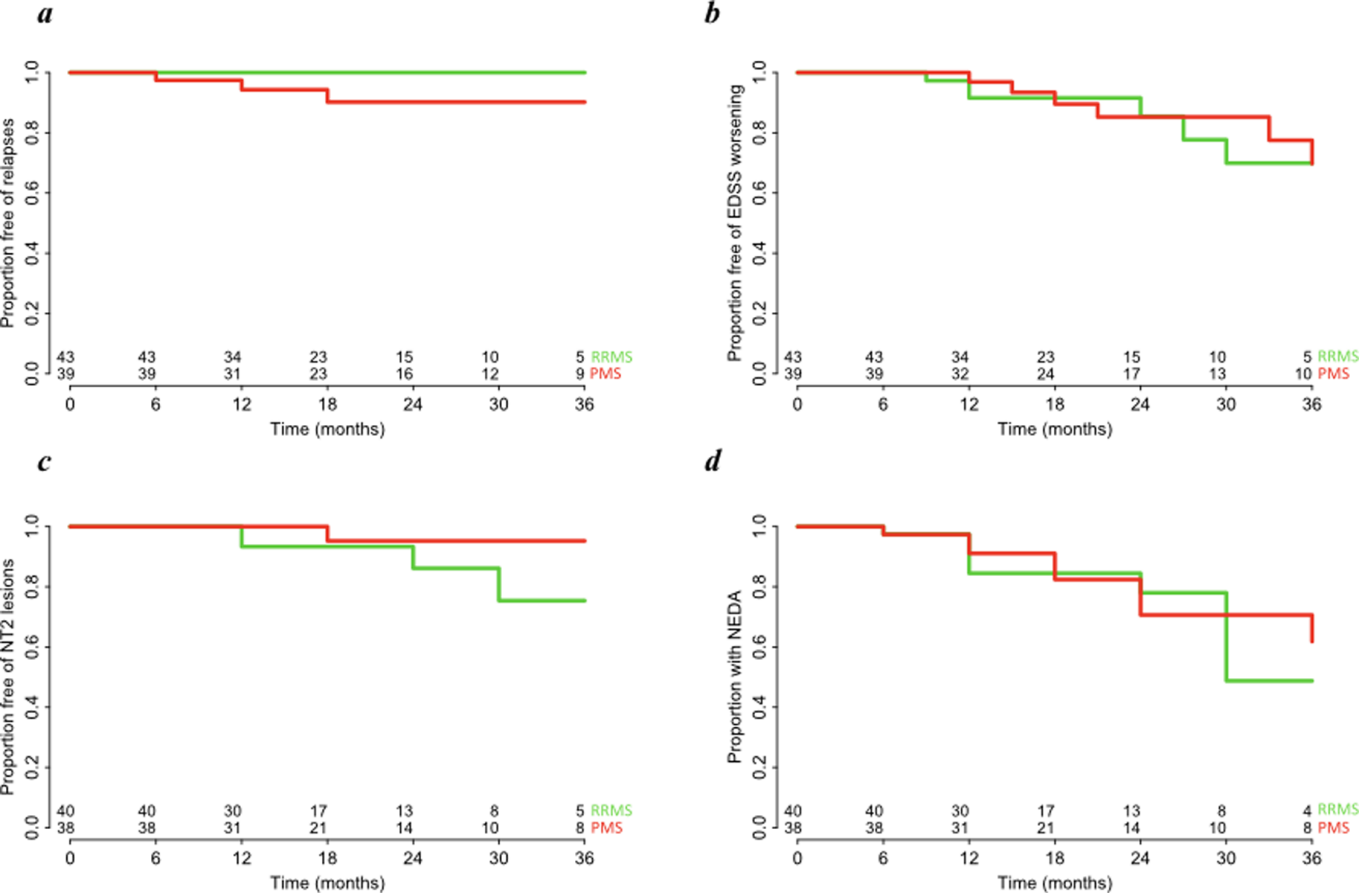
Proportion of patients free of relapses (a), free of EDSS worsening (b), free of NT2 lesions (c) and with not evidence of disease activity (NEDA) (d) during 36 months of follow up in RRMS (green) and PMS patients (red) under treatment with RTX.

The overall number of observed relapses during RTX treatment was 3 and all of them have occurred in cases of PMS at 6, 12 and 18 months since RTX initiation (Fig 1A). As expected due to the low number of relapses, there was no statistically significant difference between RRMS and PMS (*p* = 0.99). EDSS worsening was observed in 7 (16.3%) RRMS and 8 (20.5%) PMS patients (Fig 1B), also with no statistically significant difference between the two groups (HR = 0.87, 95%CI = 0.31–2.43, *p* = 0.79). The median time between baseline and the last follow up brain MRI was 12 (10–24) months for RRMS and 18 (12–29) months for PMS. A follow-up brain MRI was not available only in 3 (7.0%) RRMS and 1 (2.6%) PMS patients (presence of a pacemaker [n = 1], short follow-up [n = 3]). NT2 lesions occurred in 5 (12.5%) RRMS and 1 (2.6%) PMS patients, with no statistically significant difference between them (HR = 0.15, 95%CI = 0.02–1.31, p = 0.09) (Fig 1C). Brain GE lesions were observed only in one RRMS and one PMS patients during follow-up (at 6 and 12 months since baseline, respectively) (HR = 0.99, 95%CI = 0.06–15.88, p = 0.99). The proportion of patients without EDA status at the end of follow-up was 59 (75.6%) overall, 31 (77.5%) in RMS and 28 (73.7%) in PMS (Fig 1D), with no significant difference between groups (HR = 0.80, 95%CI = 0.32–2.03, p = 0.64). Age, sex, previous treatment with NTZ, baseline EDSS and number of relapses in the 2 years preceding RTX treatment were tested for association with risk of EDA status and no significant associations were detected (Table 2).

**Table 2:** Univariate and multivariate cox regression models testing associations between baseline characteristics and risk of EDA during RTX treatment.

Variables		Univariate analysis			Multivariate analysis		
		HR	95% CI	P	HR	95% CI	P
**Disease course**		-	-	-	-	-	-
		0.97	0.39–2.41	0.95	0.73	0.22–2.49	0.62
**Age**	** **	0.98	0.94–1.03	0.42	0.96	0.91–1.01	0.13
**Sex**	**Female**	-	-	-	-	-	-
	**Male**	2.19	0.84–5.68	0.11	2.33	0.84–6.47	0.10
**BMI**	** **	0.99	0.94–1.05	0.75	0.98	0.90–1.08	0.72
**NTZ as last DMT**	**No**	-	-	-	-	-	-
	**Yes**	0.74	0.21–2.55	0.63	0.85	0.19–3.83	0.83
**Baseline EDSS**	** **	1.03	0.80–1.33	0.80	1.1	0.80–1.50	0.56
**Brain NT2 at baseline MRI**	**No**	-	-	-	-	-	-
	**Yes**	0.96	0.79–1.17	0.70	0.97	0.77–1.21	0.76
**Relapses in 2 yrs pre-RTX**		-	-	-	-	-	-
		0.58	0.25–1.39	0.22	0.58	0.22–1.54	0.28

### Effectiveness of RTX compared to NTZ in RRMS

A total of 83 RRMS patients treated with NTZ were included in the analysis since 2007 (62.7% females, median age at NTZ start = 36 (30–41) years). Most of these were switched to NTZ from injectable DMTs (interferons and glatiramer acetate) because of disease reactivation (n = 65 [78.3%]). The median EDSS at NTZ start was 2.5 (2.0–3.5) and relapses had occurred in the 2 years preceding NTZ in 75 (90.4%) of the patients (S1 Table).

**Fig 2:**
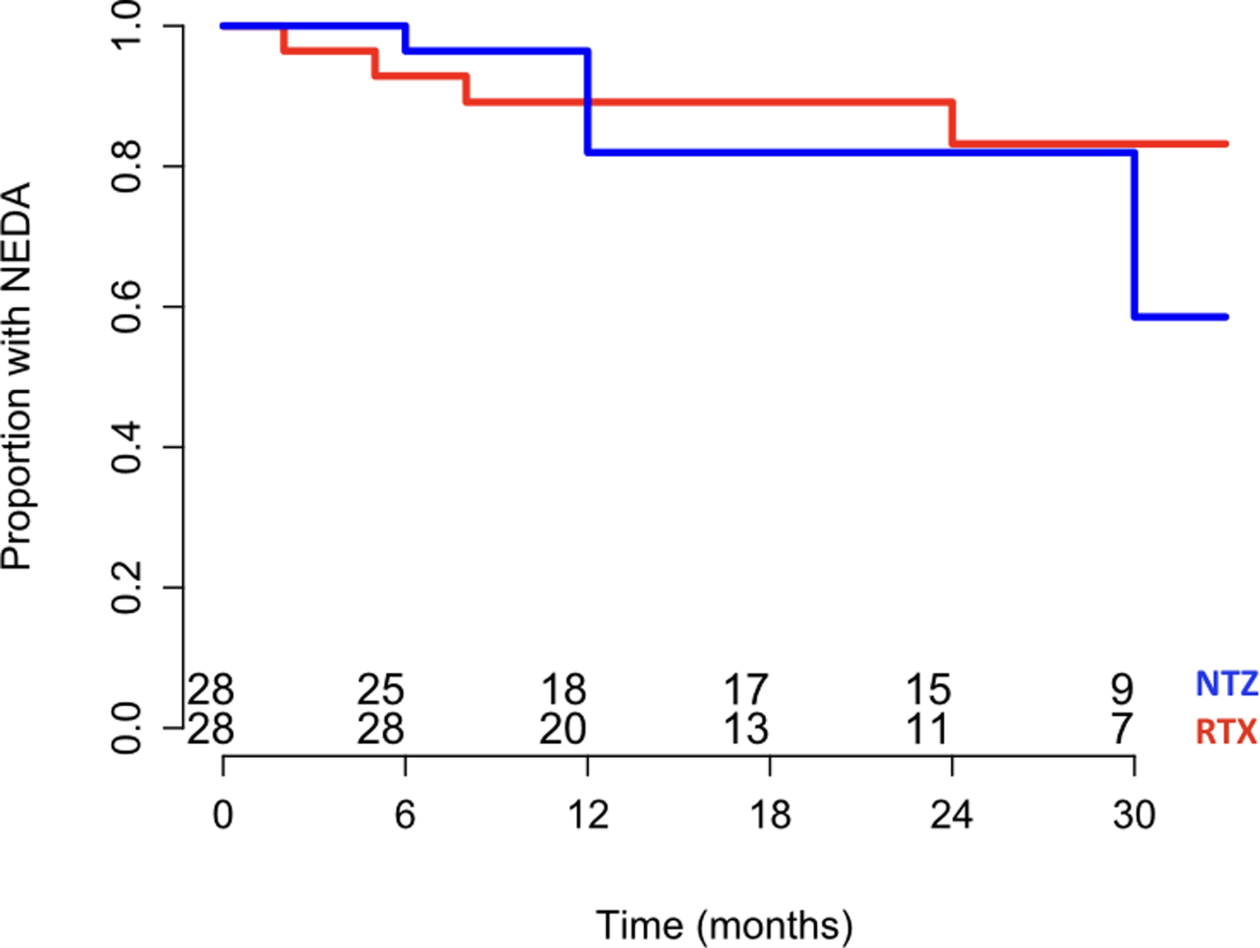
Proportion of RRMS patients with not evidence of disease activity (NEDA) under treatment with RTX (red) and NTZ (blue) over 30 months follow up.

The median follow up after NTZ start was 2.3 (1.5–3.2) years with a median of 27 (19–38) NTZ infusions. All patients had at least one brain MRI over follow-up and the median time between baseline and last available scan was 23 (14–34) months. Acute relapses, EDSS worsening and NT2 brain lesions were observed during follow-up in 2 (2.4%), 5 (6.0%) and 6 (7.2%) patients, respectively. Seventy-two (86.7%) patients had not EDA at the end of follow-up. There was no significant difference between RRMS patients under RTX and those under NTZ in terms of time to EDA after correction for age, sex, baseline EDSS, NT2 lesions at baseline scan and number of relapses in the 2 years preceding treatment (HR = 1.80, 95%CI = 0.65–5.01, p = 0.26). After matching by propensity scores, 28 pairs of RTX and NTZ treated individuals were identified with comparable baseline characteristics (S1 Fig). Similarly to the previous multivariate regression model, no significant difference was observed between RTX and NTZ in time to EDA (HR = 1.64, 95%CI = 0.46–5.85, p = 0.44) (Fig 2).

### Changes in total leucocytes, CD8+ T, CD4+ T and CD19+ B cell counts under RTX

**Fig 3:**
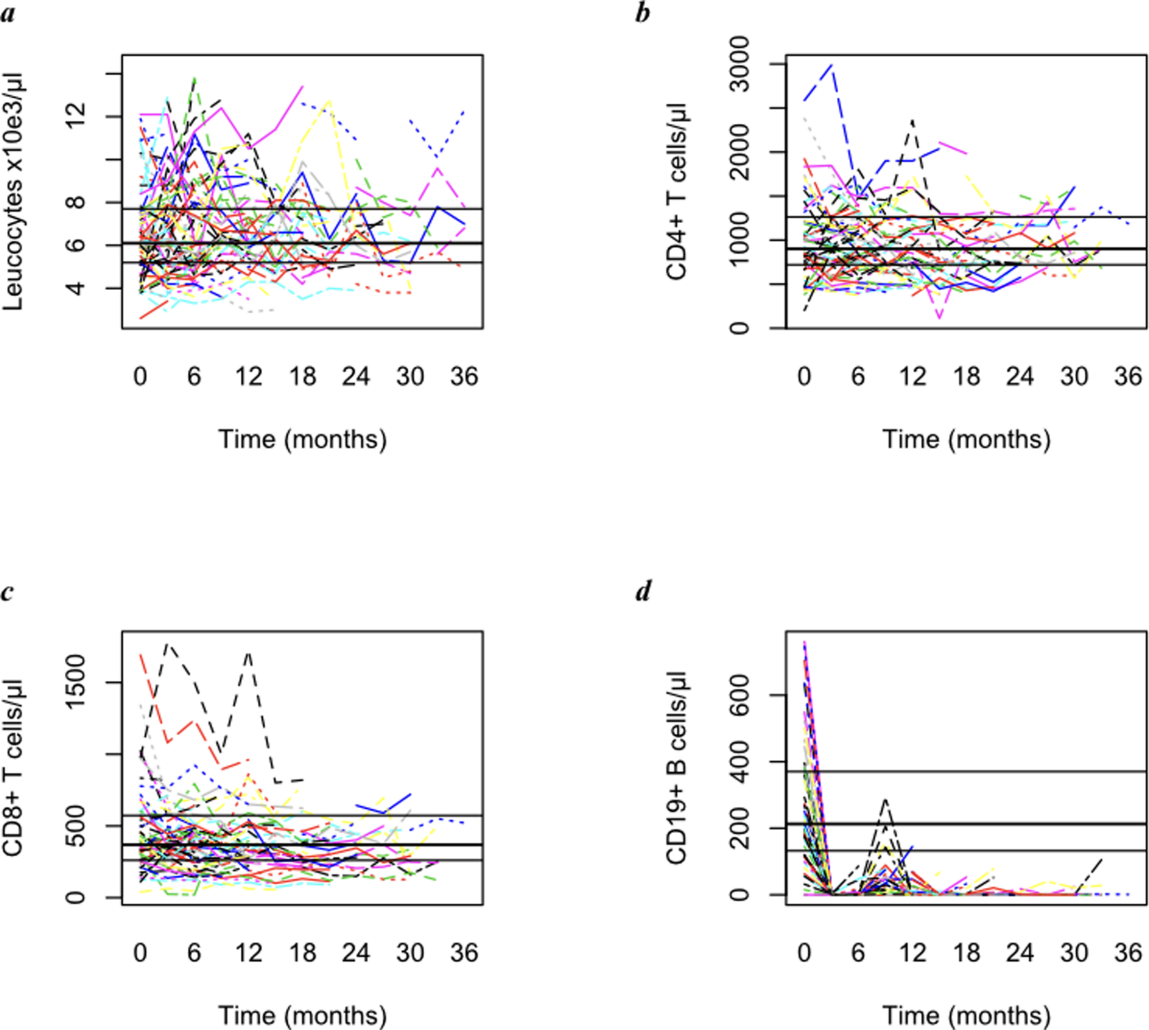
Total leucocyte (a), CD4+ T cell (b), CD8+ T cell (c) and CD19+ B cell (d) peripheral blood counts for each individual from baseline up to the 36^th^ month of follow-up during RTX treatment. Horizontal lines indicate median and IQR cell concentration at baseline (i.e. before first RTX infusion).

The median total leucocyte count at baseline was 6,100 (5,200–7,700)/μl. Leucocyte counts were averaged within each individual up to the 36^th^ month of follow-up, with a median value of 6,200 (5,400–7,600)/μl (Fig 3A). The median CD4+ T cell concentration before first RTX infusion was 902.0 (720.0–1,263)/μl, and 871.0 (690.9–1,554.2)/μl over 36 months (Fig 3B). The median CD8+ T cell count before first RTX infusion was 369.0 (261.0–572.5)/μl and 331.5 (258.5–469.5)/μl over 36 months (Fig 3C). CD19+ B cell concentration dropped from 213.0 (132.5–370.5)/μl at baseline to a median follow-up value of 0.5 (0.0–1.5)/μl (*p*<0.0001) (Fig 3D).

### Adverse events and RTX discontinuation

The total number of observed infusion related AE that were clinically significant according to treating neurologist was 10 out of 339 RTX infusions (four fever, one myalgia, two nausea, one tachycardia and two swelling of buccal mucosa). Non-infusion related AE were observed during follow-up in 11 (25.6%) RRMS (7 respiratory tract infections, 6 urinary infections, 2 shingles, 2 herpes simplex, 1 conjunctivitis and 1 vaginal yeast infection) and 13 (33.3%) PMS patients (13 urinary infections, 3 herpes simplex, 2 respiratory tract infections, 1 shingles, and 1 cytomegalovirus proctitis). Ten patients were switched from a maintenance regimen of 1,000 mg / 6 months to 500 mg / 6 months because of recurrent infections. Nine out of 82 MS patients (11.0%, 1 RRMS, 8 PMS) discontinued RTX between 1 and 94 months after the first RTX infusion. Reasons for discontinuation were disability progression in 3 PMS patients and recurrent (mainly urinary and respiratory) infections in the remaining 6 patients. The available follow-up of these patients after RTX ranges between 10 and 96 months (mean 40 months). Two patients were switched to mitoxantrone (all PMS), one to ocrelizumab (PMS), one to teriflunomide (PMS), while the remaining 5 patients (4 PMS and 1 RRMS) have not initiated any other treatment after RTX discontinuation. EDSS worsening has occurred after RTX discontinuation in 4 patients (all PMS, 1 on mitoxantrone, 3 with no treatment after RTX).

## Discussion

Encouraging positive results on effectiveness of B-cell-depleting therapies are increasingly perceived as an important addition to the existing panel of DMT in MS. Recent observational studies have indeed confirmed RTX is effective in RRMS with a discontinuation rate that is lower than that of other DMT [9]. Despite this and the positive results of phase II trials, RTX is not currently approved for the treatment of MS and can only be used as an off-label treatment option [14]. We report additional data from the largest collection of MS patients treated with RTX within Switzerland in a clinical practice setting, providing additional evidence that RTX is effective and relatively safe in this highly disabling condition.

In this study including RRMS and PMS followed for a median time of 1.5 years, almost 80% of the patients had not EDA, with no difference between RRMS and PMS. Effectiveness did not appear to be influenced by demographic and clinical characteristics at baseline, including EDSS and number of clinical relapses in the two years preceding RTX initiation, suggesting that most patients can benefit from such treatment. Only three patients experienced minor relapses and all of them had PMS. Despite the relatively small number of patients, this finding may indicate a higher efficacy of RTX when administered in the earliest stages of disease, when the inflammatory component is prominent [10]. Notably, no relapses occurred in the 16 MS patients who were switched from NTZ to RTX because of positive JCV serology. NTZ treatment is known to be associated with a potential risk of disease rebound following its discontinuation, particularly in highly active patients [6,15]. As previously suggested, these findings indicate that RTX may represent a valid treatment option also in this context [16].

Within RRMS patients, disease activity was similarly reduced in RTX vs NTZ treated individuals, both in multivariate Cox models and after propensity score based matching, further supporting a comparable efficacy between these two monoclonal antibodies [5]. This finding is in line with the recent study by Granqvist et al. reporting a superior effectiveness of RTX compared to injectable DMTs and dimethyl fumarate, and a trend towards a lower relapse rate as compared to NTZ and fingolimod in newly diagnosed RRMS [5].

The number of observed infusion related clinically significant AE was in our experience low (10 out of 339 infusions) and these were generally of mild intensity. We instead observed in some patients recurrent infections, mainly involving the urinary and respiratory tracts, requiring RTX discontinuation in 6 individuals. Similarly to ocrelizumab registration trials (OPERA [17] and ORATORIO [18]), we found that infections were more frequent in progressive patients, suggesting RTX should be used with caution and being vigilant for the potential occurrence of severe infections particularly in fragile patients [19]. No cases of progressive multifocal leucoencefalopahy or cancer were detected. In line with this, no serious concern for increased risk of cancer has arisen from RTX use in different clinical indications for almost two decades [20]. As expected, there was a striking and stable reduction of CD19+ B cell concentration after RTX initiation, while CD4+ and CD8+ levels did not appear considerably influenced by RTX treatment.

The main limitation of this study is represented by its retrospective design and the relatively small number of patients. However, we made use of data that are routinely and prospectively collected at our MS center as part of daily clinical practice. Our center also represents the only tertiary care neurological clinic providing treatment with monoclonal antibodies in MS within Southern Switzerland. We therefore believe the individuals included in this study are highly representative of the overall patient population. We also limited the potential effect of confounding factors by using multivariate adjusted regression models and by appropriately matching RTX with NTZ treated patients by propensity scores with stringent criteria [21,22].

Another limitation is represented by the mixed doses of RTX being used in this study. Most of the patients were treated with a RTX 1,000 mg every 6 months, but some were switched to a reduced dosage (500 mg every 6 months) because of variable reasons (e.g. recurrent infections, low body weight), as judged by the treating physician. We believe our sample size was too small to investigate a potential difference in treatment efficacy between these two doses. However, another recent retrospective study of larger sample size from Sweden found no major differences in RTX efficacy between 1,000 and 500 mg every 6 months [9]. Anti-RTX antibodies were not measured being their relevance still controversial [5,23].

To conclude, this study provides additional evidence for the use of RTX in MS, with a comparable effectiveness to that of NTZ within the relapsing-remitting subtype and a potential benefit also in progressive cases. RTX could represent an additional effective, relatively cheap and safe therapy in the panel of existing MS treatments.

## Supporting information

S1 Table Baseline characteristics at first NTZ infusion in the 83 MS patients included in the study. Continuous and ordinal variables are described by median (IQR), categorical variables by counts and percentage.
(PDF)

S1 Fig Baseline characteristics (age, sex, EDSS, number of relapse prior to treatment and brain T2 lesion load) in the original sample of NTZ and RTX treated RRMS patients and after matching by propensity scores. (PDF)
