# Supplementary material for: Effectiveness and safety of Rituximab in multiple sclerosis: an observational study from Southern Switzerland
Source: PLoS One. 2018 May 14;13(5):e0197415. doi: 10.1371/journal.pone.0197415 (PMC5951582; doi:10.1371/journal.pone.0197415)
Supplement: S1 Table — Continuous and ordinal variables are described by median (IQR), categorical variables by counts and percentage. (PDF) [file pone.0197415.s001.pdf]

S1 Table. Baseline characteristics at first NTZ infusion in the 83 MS patients included in the study. Continuous and ordinal variables are described by median (IQR), categorical variables by counts and percentage.

Variables		Median (IQR) /count (%)
**Age**		36 (30-41)
**Sex**	M	31 (37.3)
	F	52 (62.7)
**EDSS**		2.5 (2.0-3.5)
**Number of relapses 2 yrs pre-NTZ**	0	8 (9.6)
	1	31 (37.3)
	2	30 (36.2)
	3	8 (9.7)
	4	3 (3.6)
	5	1 (1.2)
	6	2 (2.4)
**Brain T2 lesions**	0-1	0 (0)
	1-9	8 (9.6)
	>9	75 (90.4)
**Brain GE lesions**	No	60 (72.3)
	Yes	23 (27.7.0)
**Number of DMTs before NTZ**	0	15 (18.1)
	1	47 (56.6)
	2	12 (14.5)
	3	4 (4.8)
	4	2 (2.4)
	5	3 (3.6)
**Last treatment before NTZ**	Interferon	52 (62.6)
	Glatiramer acetate	13 (15.7)
	None	14 (16.9)
	Other	4 (4.8)
