# Supplementary material for: Effectiveness and safety of Rituximab in multiple sclerosis: an observational study from Southern Switzerland
Source: PLoS One. 2018 May 14;13(5):e0197415. doi: 10.1371/journal.pone.0197415 (PMC5951582; doi:10.1371/journal.pone.0197415)

S1 Fig. Baseline characteristics (age, sex, EDSS, number of relapse prior to treatment and brain T2 lesion load) in the original sample of NT[illegible] and RTX treated RRMS patients and after matching by propensity scores.

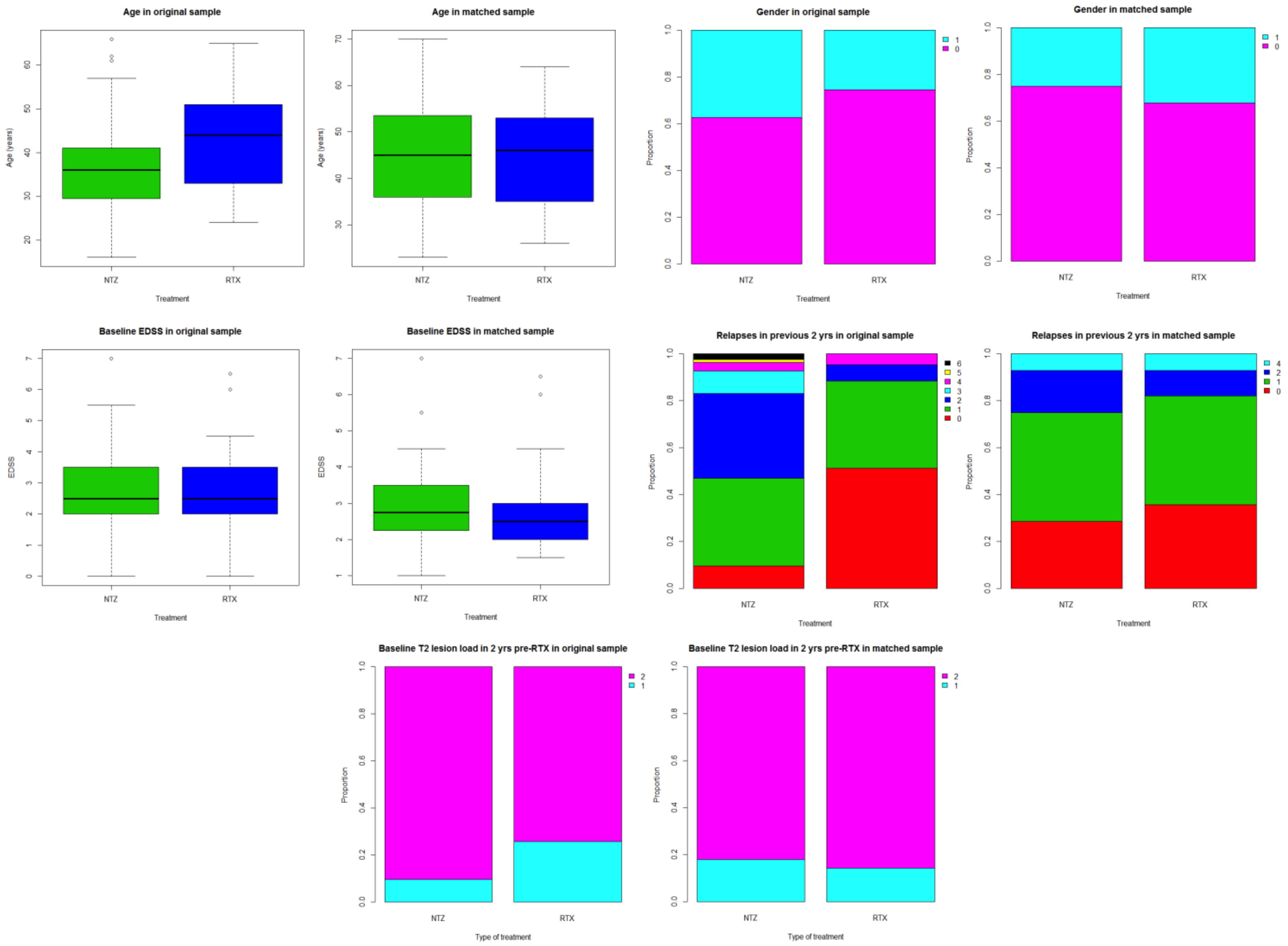

Supplement: S1 Fig — (PDF) [file pone.0197415.s002.pdf]
